# Managing menopause after cancer: a qualitative analysis of healthcare professionals’ perspectives

**DOI:** 10.1007/s11764-026-02042-0

**Published:** 2026-05-18

**Authors:** Dorcas Serwaa, Shanton Chang, Lewis Gauci, Michael Jefford, Carolyn Ee, Martha Hickey, Kate Rolshoven, Jennifer L. Marino, Paul A. Cohen, Christobel Saunders, Nipuni Susanto, Michelle Peate

**Affiliations:** 1 Department of Obstetrics, Gynaecology and Newborn Health, Faculty of Medicine, Dentistry and Health Sciences, The University of Melbourne, Melbourne, VIC Australia; 2 Department of Obstetrics, Gynaecology and Newborn Health, The Royal Women’s Hospital, Melbourne, VIC Australia; 3 School of Computing and Information Systems, The University of Melbourne, Melbourne, VIC Australia; 4 Rural Clinical School, Department of Rural Health, Melbourne Medical School, University of Melbourne, Melbourne, VIC Australia; 5 Department of Medical Oncology and the Australian Cancer Survivorship Centre, Centre for Health Services Research in Cancer, Peter MacCallum Cancer Centre, Melbourne, VIC Australia; 6 Sir Peter MacCallum Department of Oncology, University of Melbourne, Melbourne, VIC Australia; 7 Caring Futures Institute, College of Health and Enablement, Flinders University, Bedford Park, South Australia Australia; 8 Consumer Representative, Department of Obstetrics and Gynaecology, Royal Women’s Hospital, University of Melbourne, Melbourne, VIC Australia; 9 Specialty of Child and Adolescent Health, Sydney Medical School, Faculty of Medicine and Health, The University of Sydney, Sydney, NSW Australia; 10 Division of Obstetrics and Gynaecology, Medical School, The University of Western Australia, Perth, Australia; 11 Melbourne Medical School, The University of Melbourne, Melbourne, VIC Australia; 12 Department of Surgery, The Royal Melbourne Hospital, Melbourne, VIC Australia

**Keywords:** Menopause, Menopause management, Cancer survivors, Healthcare professionals, Menopause perspective

## Abstract

**Background:**

Menopausal symptoms after cancer (MSAC) are common and can affect overall quality of life. Cancer survivors experience a variety of symptoms for which they may seek support from healthcare professionals (HCPs). Yet, managing MSAC may be challenging for some HCPs. Therefore, understanding HCPs’ perspectives and challenges in providing MSAC care could help propose solutions to improve care provision.

**Methods:**

Using semi-structured, one-on-one interviews, we explored the perspectives and challenges of 20 HCPs from a range of specialties involved in the care of women with MSAC. Interviews were transcribed and analyzed using a reflexive thematic approach with NVivo v12.

**Results:**

Twenty HCPs aged 37–68 years were interviewed. The sample comprised females (*n* = 17), with primary care physicians being the largest HCP grouping (*n* = 6), of whom half were MSAC specialists. Participants’ clinical experience ranged from 1 to 35 years (19.95 ± 9.88), and their experience in managing MSACs ranged from 3 to 35 years (14.25 ± 8.69). Five themes with 10 subthemes were identified: (1) contested view of menopause after cancer; (2) HCPs’ resilience in patient-centred care; (3) multidisciplinary patient care; (4) trial, adaptation, and the search for symptom relief; and (5) care constrained by uncertainty and risk.

**Conclusion:**

HCPs recognized the substantial burden of MSAC and have adopted patient-centred care and multidisciplinary approaches. However, the confidence in prescribing menopausal hormone therapy was identified as an area for improvement in clinical practice. For all categories of HCPs, upskilling through targeted education and decision-support tools may improve care.

**Implications for Cancer Survivors:**

For cancer survivors, these findings indicate that HCPs recognise the burden of menopausal symptoms after cancer and that improvements in clinician education, decision‑support tools and clearer referral pathways should translate into timelier, evidence‑informed care (including considered use of MHT where appropriate), better multidisciplinary support and shared decision‑making — all of which aim to reduce symptom burden and improve quality of life.

**Supplementary Information:**

The online version contains supplementary material available at https://doi.org/10.1007/s11764-026-02042-0.

## Introduction

Advances in cancer care have led to a growing number of survivors who live with the short- and long-term consequences of the disease and its treatment [1, 2]. A commonly reported consequence of treatment in women, predominantly breast cancer survivors, is treatment-related menopause [3, 4]. Between 63 and 89% experience menopausal symptoms such as hot flushes and night sweats [5–7], 51 and 75% vaginal dryness [7–9], 86 and 89% sleep difficulties [7, 8], and 62% mood disturbance [7]. If left untreated, these symptoms can cause distress [1], can provoke feelings of helplessness [10], can impact interpersonal or intimate relationships and work [11–13], may adversely affect overall quality of life (QoL) [1, 3, 11], and can also lead to non-adherence to cancer treatments such as endocrine therapy [14].

Management of menopausal symptoms is an important concern for women who have experienced cancer. Several studies identify these among the top (“prioritized”) unmet needs of cancer survivors [1, 15, 16]. Effective interventions are available, including menopausal hormone therapy (MHT) [17, 18], non-hormonal medications (e.g. some classes of antidepressants, anticonvulsants, anti-hypertensives) [19, 20], and complementary therapies (e.g. clinical hypnosis, and cognitive behaviour therapy (CBT)) [19]. However, data from women who have experienced breast cancer indicate that fewer than 32% of those who reported needing treatment were offered options [7, 14, 21], and 26–60% expressed the need for greater support to manage menopausal symptoms [7, 22, 23].

Women experiencing menopausal symptoms after cancer treatments are seen in primary and tertiary care settings by a diverse range of HCPs with varying expertise, including primary care physicians, endocrinologists, oncologists, surgeons, cancer nurses, and gynaecologists [12, 24]. Evidence from general menopause care indicates that some HCPs are uncomfortable discussing menopause and lack confidence in managing core aspects of care, including prescribing MHT [25, 26]. Training gaps are likely a key contributor. Among US resident doctors, 20% reported no menopause-related training, and 93% felt inadequately prepared to manage menopausal symptoms [26]. These findings highlight an important HCP-level factor that may contribute to patients’ unmet need for additional information and support.

In Australia, only one qualitative study has explored the management of menopause among general practitioners, gynaecologists, and pharmacists; it concluded that participants had a good understanding of menopause, were informed about its implications, and cautiously supported the use of MHT [27]. This study focused on menopause in the general population rather than in survivors, where contraindications and additional complexities are more common. It also did not include cancer-specific HCPs.

Despite the high number of women who require HCPs’ services for post-cancer menopausal symptoms management, to the best of our knowledge, there is no evidence on how HCPs manage menopause in women who have experienced cancer. It is therefore imperative to explore the views, practices, and strategies of a diverse range of HCPs involved in the care of these women. The aim of the current study was to explore HCPs’ experiences and perspectives on the management of menopause in women who have experienced cancer. These findings will highlight the nature of care HCPs provide and the challenges they face, and inform the development of relevant resources to meet survivors’ needs.

## Method

### Study design

We adopted an interpretivist and pragmatic orientation. From the interpretive worldview, we treated participants as active contributors to our understanding of how menopause after cancer is managed, recognizing that their accounts could be shaped by professional role, clinical experience, and the social and organizational contexts in which they practice [28, 29]. The pragmatic worldview informed the use of qualitative design to allow in-depth probing of each participant’s views [30].

### Study setting and ethical considerations

The study was conducted between October 2023 and April 2024 at the Department of Obstetrics, Gynaecology and Newborn Health, University of Melbourne, and the Royal Women’s Hospital. Ethics approval was obtained from the University of Melbourne (HREC no. 2023–26437–44192–3), and participants provided written informed consent. The methodology and results are reported in accordance with the Consolidated Criteria for Reporting Qualitative Research (COREQ) [31].

### Participants and recruitment

HCPs managing menopause after cancer were invited through advertisements in e-newsletters and on the social media channels of various Australian HCP societies. Invitation targeted primary care physicians, nurses, oncologists, gynaecologists, endocrinologists, menopause specialists, and pharmacists. Interested HCPs completed a pre-interview survey containing an eligibility checklist, an informed consent sheet, and baseline information. To participate, HCPs were required to hold a current registration and a valid license from the Australian Health Practitioner Regulation Agency (AHPRA); be in active clinical practice; have at least 1 year of experience in managing menopause after cancer; reside in Australia; and have the capacity to consent. We used purposive sampling to ensure representation by age, gender, specialty (e.g. primary care, endocrinology), years of clinical experience, and experience managing menopause after cancer.

### Data collection

Interviews were conducted through Zoom video conferencing, an approach shown to be as effective as face-to-face interviews [32]. This also enabled sample diversity across Australia. Three authors developed the semi-structured interview guide, which was reviewed and refined based on all authors (Additional material 1). The guide explored the participants’ perceptions and involvement in managing menopausal symptoms in cancer survivors (e.g. “Please can you describe what your role has been in the management of cancer survivors’ menopausal symptoms?”), the symptoms they managed (e.g. “What symptoms do you see more often? How does it impact these women’s lives?”), and their usual management approaches (e.g. “What management options do you offer these patients?”).

All interviews were conducted by DS, a female PhD candidate (MSC, BSc (Hons)), trained in qualitative methods. DS had no clinical role in menopause care and approached the study as a researcher focused on service improvements. Apart from menopause specialists (MH and PC), who may have had professional relationships with some MSAC participants, no other team members knew the participants. All interviews were conducted between DS and each participant, with no additional attendees present. The interviews were audio‑recorded and transcribed verbatim. No interviews were repeated, and field notes were taken during the interviews. Each participant was offered a gift card ($80) in appreciation of their time. Recorded interviews were transcribed verbatim using Trint software [33]. The transcripts were not returned to participants to provide comments and/or feedback, but DS checked for accuracy and anonymity.

### Analysis

Transcripts were imported into NVivo version 12 and analyzed iteratively alongside data collection, and a reflexive thematic analysis was used based on Braun and Clark’s six principles [34]. DS conducted inductive coding and theme development. Familiarization with the dataset was achieved through repeated viewing and listening to each video recording, and reading the transcripts to identify patterns, emotions, tone, and salient issues. Codes were generated by labelling statements relevant to the study’s objective with a few words or short phrases that captured their meaning. Some statements were assigned one or more codes. Where necessary, codes were revised to ensure clarity and consistency, and to limit the number of unique codes. The codes and their associated statements were merged into subthemes and further organized into themes. Each theme was reviewed against the dataset, initial codes, and subthemes, and refined when necessary.

Short definitions summarizing the core idea of each theme were written, which were then given interpretive names. Recruitment ceased when information redundancy was reached; after analyzing 12 transcripts, three additional transcripts yielded repeated information [35]. To ensure perspectives from HCPs not yet captured, four additional interviews were conducted (with a pharmacist, radiation oncology nurse, and gynaecological oncologist), but no new information was identified. DS, SC, and MP determined that the data were adequate in terms of richness, addressed the research question, and that purposive sampling ensured the intended diversity of the HCPs [34]. The findings were not returned to the participants to provide their feedback.

### Reflexivity statement

DS held regular meetings with SC and MP for reflexive dialogue to discuss emerging themes and definitions, resolve discrepancies, and reflect on DS’s assumptions and potential biases, ensuring that participants’ voices remained central to the interpretation [36, 37]. The final write-up integrated subthemes, themes, and definitions, and used exemplar quotations to support interpretations, ensuring transparency [38]. To enhance credibility, we triangulated the findings across existing literature, and we demonstrated dependability through ensuring that information redundancy was achieved [38]. The multidisciplinary team included experts in psycho-oncology, information technology, primary care, oncology, gynaecology, menopause after cancer care, implementation science, and a consumer representative. To reduce confirmatory bias, senior menopause clinicians, primary-care physicians, and cancer clinicians who contributed to developing the interview guide were not involved in the analysis or interpretation of the data [39].

## Results

### Sample

**Fig. 1:**
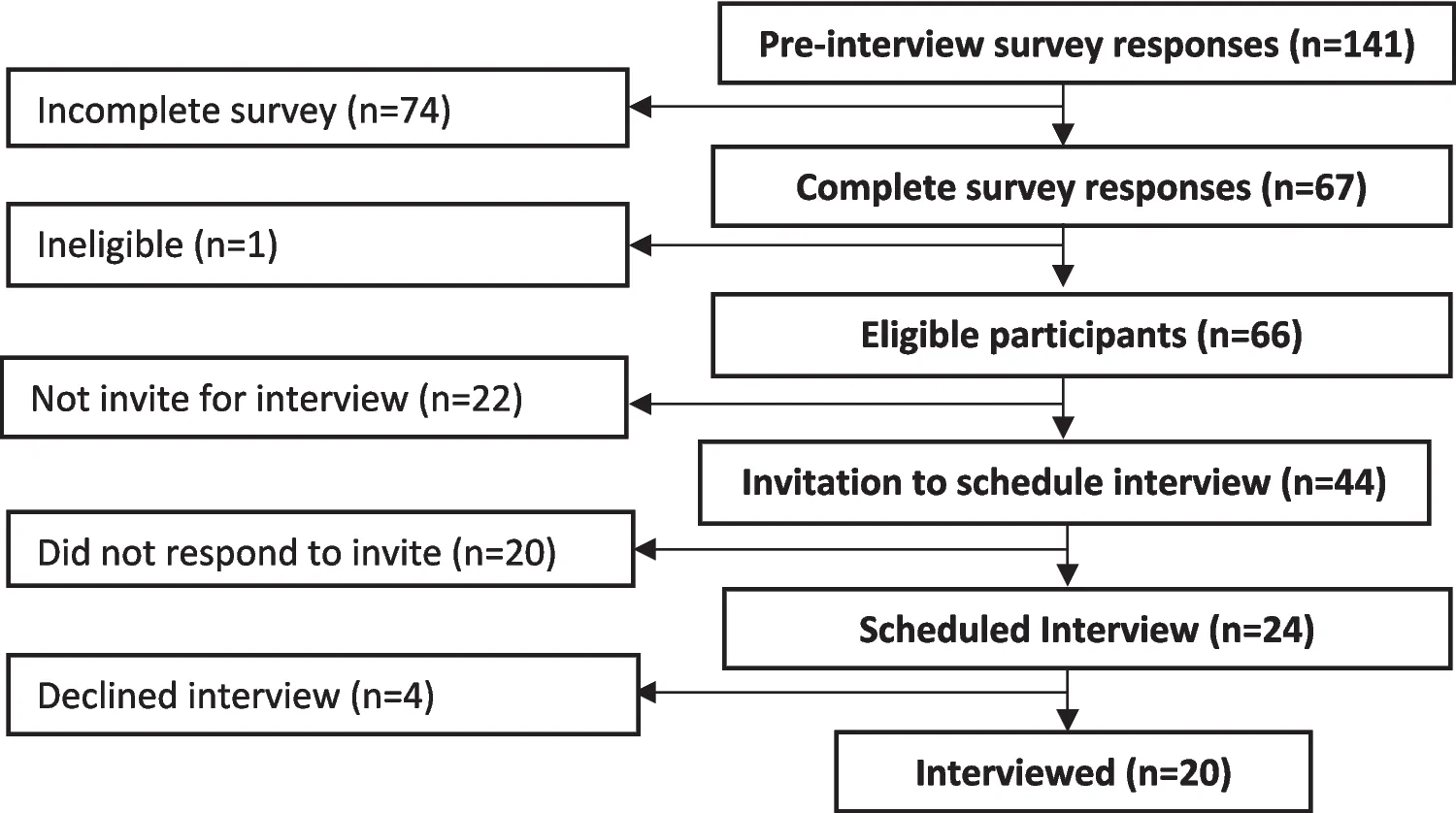
Recruitment process of study participants

The sampling process is shown in Fig. 1. Of 141 potential HCPs who expressed interest, 67 completed the pre-interview survey. One physiotherapist was excluded due to a lack of relevant experience. Forty-four HCPs were purposefully invited for interviews to ensure diversity (age, gender, speciality, years of practice, and menopause after cancer experience), while 23 HCPs (mostly primary care physicians and breast care nurses) were thanked but not invited. There were no differences in age, gender, years of clinical practice, and years of MSAC experience between those invited and not invited. Of the 44 invited, 20 did not respond (no reason given), 24 were scheduled, four declined due to time constraints, and 20 were interviewed. The interviewed group (*n* = 20) did not differ from those who declined or did not respond (*n* = 24); the latter were predominantly female primary care physicians and breast care nurses with 11–20 years of practice, a characteristic represented among the HCPs interviewed. Interviews averaged 30 min (ranging from 25 to 35 min).

### Sociodemographic and professional characteristics of study participants

Table 1 presents participants’ baseline characteristics. Participants’ age ranged from 37 to 68 years (53.75 ± 9.36) and most identified as female (*n* = 17). The majority were born in Australia (*n* = 15), lived in urban areas (*n* = 15), were based in Victoria (*n* = 12), and were employed part-time or casually (*n* = 11). The most common specialty was primary care physician (*n* = 6), of whom half (*n* = 3) practized as MSAC specialists. Overall clinical experience ranged from 3 to 35 years (mean, 19.95 ± 9.88), with experience managing menopause after cancer ranging from 3 to 35 years (mean, 14.25 ± 8.69). Half reported that fewer than 20% of the patients they saw fortnightly were being managed for menopausal symptoms after cancer. Hot flushes (*n* = 20) and vulvovaginal dryness (*n* = 19) were the most frequently managed symptoms.

**Table 1:** Sociodemographic and professional characteristics of study participants

Variables	Category	*N* = 20
Age (years)	Range: Min = 37, Max = 68; mean: 53.75 ± 9.36	
Gender	Female	17
	Male	< 5
Country of birth	Australia	15
	Other	5
Ethnicity	White	9
	Asians	< 5
	Not specified	8
State	Victoria	12
	New South Wales	< 5
	Western Australia	< 5
	South Australia	< 5
	Queensland	< 5
Geographical location	Urban	15
	Rural/regional	5
Employment status	Employed full-time	9
	Employed part-time/casual	11
Specialty	Primary care physicians^a^	6
	Breast care nurse	< 5
	Pharmacist	< 5
	Endocrinologist^a^	< 5
	Breast surgeon	< 5
	Gynaecologist	< 5
	Radiation oncology nurse	< 5
	Menopause clinical nurse consultant	< 5
	Medical oncology nurse practitioner	< 5
	Gynaecological oncologist	< 5
	Clinical nurse specialist	< 5
Clinical experience (years)	Min = 1, Max = 35 (19.95 ± 9.88)	
	1–10	5
	11–20	< 5
	> 20	11
Clinical experience in managing menopause in cancer survivors (years)	Min = 3, Max = 35 (14.25 ± 8.69)	
	1–10	7
	11–20	8
	> 20	5
Proportion of patient cohort managed fortnightly for menopausal symptoms after cancer	< 20%	10
	20–39%	7
	≥ 40%	< 5
Kinds of symptoms managed (*n* = yes)	Hot flushes	20
	Night sweats	18
	Vulvovaginal dryness	19
	Vulvovaginal discomfort/irritation	13
	Pain during sex	13
	Discomfort/irritation when urinating	5
	Other	11

Counts fewer than 5 are suppressed and shown as “ < 5” to protect privacy and prevent potential re-identification of participants

^a^Also worked as menopause symptoms after cancer specialist

### Themes, subthemes, and sample quotes identified in the study

Five themes and 10 subthemes were identified. The themes were (1) contested views of menopause after cancer; (2) HCPs’ resilience in patient-centred care; (3) multidisciplinary patient care; (4) trial, adaptation, and search for symptom relief; and (5) care constrained by uncertainty and risk (Table 2).

**Table 2:** Themes and sub-themes of HCPs’ perspective in managing menopausal symptoms in women who have experienced cancer

Themes	Sub-themes	Example quotes
Contested views of menopause after cancer (compared to natural menopause) (Definition: Participant’s perceptions about severity of menopause in women who have experienced cancer compared to women who undergo natural menopause was varied)	1.1 Treatment-induced menopause was uniquely severe	“… the women who have, perhaps been sent into menopause due to their chemotherapy, they’re just automatically there and I think that they experienced sort of more severe symptoms because of that.” [Radiation Oncology Nurse 1]
	1.2 Evidence-informed perspectives on treatment-induced menopause	“It depends on who you want to compare them to, in general I’d say there’s as many bad ones to the cancer ones as there is bad ones for the normal. The normal ones might be more distressed about the hot flushes, lack of sleeping…, but the cancer one is more anxious because they’ve had a mental insult…”[Primary Care Physician 1, MSAC specialist]
2.0 HCPs’ resilience in patient-centred care (Definition: how participants assess a patient’s menopause symptoms and make decisions around treatment options)	2.1 Constructing an understanding of symptoms	“I use the menopause rating scale, obviously that help guide the conversation […] I would address each of those issues individually…” [Breast Care Nurse 1]
	2.2 Negotiated treatment decisions	“…in terms of medication, a lot of women don’t like taking medication, particularly if they’ve gone through chemotherapy. So, it’s sort of like, you know, a staged sort of approach, and discuss whether they’d prefer to try some simpler options first….” [Breast Care Nurse 3]
3.0 Multidisciplinary patient care (Definition: consultation among HCPs in managing symptoms)	3.1 Recognising knowledge gaps and referring onwards	“I refer both to the MSAC clinic* and occasionally to other menopause specialist clinics.” [Breast Surgeon 1]
	3.2 Interprofessional collaboration to managing complex cases	“…we contact their medical oncologist, after having an agreement, we can consider hormonal replacement, and we can start in our clinics.” [Primary Care Physicians 3]
4.0 Trial, adaptation, and the search for symptom relief (Definition: how participants described managing menopausal symptoms as an iterative process of trial and adjustment and how they evaluate success of treatment)	4.1 Prioritising low-risk interventions	“ … So obviously we would just go over the simple lifestyle factors that women could sort of and try and incorporate into the day to day, such as, wearing layered clothing and carrying a fun, trying to cut down on triggers like caffeine or alcohol, try to really encourage them to exercise and, to keep within a healthy weight range…, we might talk about acupuncture or, doing relaxation or meditation..” [Menopause clinical nurse consultant 1] “…I sometimes will prescribe medications such as venlafaxine, or clonidine for the symptoms…” [Breast Surgeon 1]
	4.2 Patient-informed evaluation of treatment success	“…say that in more than half of patients that I had prescribed that [venlafaxine] to will experience or report some improvement in their status…”[Medical Oncology Nurse Practitioner 1]
5.0 Constrained provision of care in the context of uncertainty and perceived risk (Definition: participant challenges in the management of symptoms in women who have experienced cancer)	5.1 Expertise and capacity constraints	“A lot of GPs, a lot of oncologists find this area very difficult. I find a lot of GP’s don’t have a lot of knowledge…” [Endocrinologist 1, MSAC specialist]
	5.2 Negotiating risk: “hesitation in prescribing MHT”	“I wouldn’t put anyone on any hormone replacement therapy, even vaginally…. my experience is that, never or close to never using hormone replacement therapy…” [Primary Care Physician 6]

*MSAC* menopausal symptoms after cancer, *MHT* menopausal hormone therapy

#### Contested views of menopause after cancer

Participants expressed varying perspectives on the severity of treatment-induced versus natural menopause. The divergence centred on whether menopause after cancer should be understood as fundamentally more severe than for women with natural menopause or as part of the shared symptom spectrum.

##### Treatment-induced menopause was uniquely severe

Some participants described treatment–induced menopause as more severe when compared with natural menopause. They perceived that, in pre- and peri-menopausal women, the oophorectomy, ovarian suppression, or aromatase inhibitor therapy causes an immediate decline in ovarian function. As a result, most participants described menopause after cancer as sudden, more intense or debilitating compared with the gradual onset of natural menopause.

…it’s not a natural menopause, so it’s quite sudden. When we treat people for cancer, it’s [menopause] pretty immediate…depending on what the treatment is or if we’re removing the ovaries…[I] feel that the symptoms are much more severe. [Menopause Clinical Nurse Consultant 1]

A number of participants described menopausal symptoms as intensified when experienced along with the psychological burden of cancer (uncertainty, trauma, anxiety, fatigue), with some referring to these symptoms as a “by-product” of cancer treatment.

…it’s obviously a mix of a whole lot of other life experiences that are really traumatic… you’re anxious, you’re tired, you’re wondering if the symptoms that you’ve gotten are related to the cancer that you’ve got, fewer therapeutic options to deal with them… [Primary Care Physician 4]

Most of these participants described their perspectives as grounded primarily in clinical experience from interacting with and managing menopause in cancer survivors and women without cancer.

##### Evidence‑informed perspectives on treatment‑induced menopause 

Some participants stated that emerging research evidence prompted them to reassess their prior beliefs that treatment‑induced menopause is invariably more severe than natural menopause and said it altered how they framed and managed symptoms.

Well, in fact, it’s only recently that we have data that has clarified to some extent that it’s not reasonable to say that all women who go through a surgical menopause have a more severe and more sudden and more intense onset of symptoms, or that they will more likely need treatment… [Primary Care Physicians 2, MSAC Specialist]

#### HCPs’ resilience in patient-centred care

Participants described various efforts to provide comprehensive, individualized care. Many reported that these efforts often relied on their personal initiative, clinical experience, and risk interpretation rather than on structured post-treatment menopause care models.

##### Constructing an understanding of symptoms

Some participants reported that menopause after cancer was complex and required in-depth assessment, including review of referrals, medical history, and comorbidities to identify contraindications. This was followed by an evaluation of menopausal symptom impact to tailor care to address the patient’s needs.

I’ll start at the referral and then depending on the type of cancer they’ve got and depending on the [menopausal] symptoms, […]. I do a full history and assessment and their past history…if it’s a cancer that you can use HRT, if they’ve had a bone marrow transplant, I will check them for bone versus graft disease […]. So go through all that symptomatology and then make a program up of what medication they might or might not need. [Primary Care Physician 3]

##### Negotiated treatment decisions 

Many other participants mentioned that treatment decision-making involved weighing the safety and available evidence alongside patients’ preferences, and that care plans were co-constructed within these parameters.

I look at what sort of cancer they’ve had, and what the evidence says regarding the safety of prescribing hormones or non-hormones… and patient preference is really important. Some want tablets, not patches or gels, and some just don’t want any medical treatment, you go with whatever they are willing to accept and tolerate… [Gynaecologist 1]

#### Multidisciplinary patient care

Participants described management of menopause after cancer as a collaborative process involving negotiation and coordination among multiple HCPs, rather than the responsibility of a single HCP group, with some treatment decisions discussed and agreed upon between HCPs.

##### Recognising knowledge gaps and referring onwards

Participants emphasised the need to work with other specialists to ensure patients received appropriate support. They mentioned that referrals were common for patients with severe symptoms or complex needs, such as those with multiple comorbidities that exceeded their scope of practice. Most participants referred to MSAC clinics or to other clinicians, including general menopause specialists, oncologists, endocrinologists, and psychosexual counsellors.

…if they’ve really struggled, I will send them to endocrinologist or to a menopause specialist or women’s health team…I feel like I’m not necessarily the best person to look at that… [Gynaecological oncologist 1]

##### Interprofessional collaboration in managing complex cases

Some participants reported that decisions around MHT were made collaboratively, typically involving the treating HCPs (most commonly primary care physicians or gynaecologists) and cancer-treatment team (most often the oncologists or breast surgeons). Many of them mentioned they deferred to, consulted with, or sought approval from the oncologists or multidisciplinary (MSAC) team before initiating MHT. Even when confident in prescribing MHT, they sought confirmation as a professional courtesy or protective measure.

…if they are non-hormonal dependent cancers, then I will often use MHT, but as a courtesy, I get the approval of the treating oncologist or surgeon, or sometimes I ask a gynaecologist if it’s safe to use hormones. So, as a [primary care physician], I do check with the treating specialist or someone who knows before I give hormones. [Primary Care Physician 5, MSAC specialist]

#### Trial, adaptation, and the search for symptom relief

Participants viewed management as a layered and iterative process involving ongoing adjustment of treatments, managing expectations, and evaluating patients’ responses. Across accounts, success was framed not as “absence of symptom”, but as incremental improvement.

##### Prioritising low-risk interventions

Non-pharmacological and/or non-hormonal pharmacological strategies were commonly positioned as the initial step, regardless of whether participants could prescribe MHT. Most mentioned non-pharmacological strategies, such as lifestyle modifications, mind-body and psychological interventions, were often introduced first.

…we do have a GP at our wellness centre who offers acupuncture, sometimes I recommend CBT… Just some practical things, reducing stress, avoiding alcohol, caffeine, certain foods…, certainly exercise… [Breast Care Nurse 1]

Participants mentioned that when symptoms persisted, non-hormonal medications were added to the non-pharmacological strategies. Some stated that their choice of medication depended on the patient’s symptom profile, comorbidities, side-effect tolerance, and their perceived responsiveness.

…I’ve tried oxybutynin, escitalopram, gabapentin, and Effexor [venlafaxine]. Effexor tends to be my second-line pharmacological after gabapentin. Unless a person has a history of anxiety and she’s not already on an anti-anxiety medication, they are the ones I find who probably respond best. Again, there can be side effects, and some of them won’t tolerate it… [Primary Care Physician 2, MSAC specialist]

Participants also described MHTs as the “gold standard”, but said their use was conditional. For instance, some mentioned they considered contraindication, and when the perceived risk of prescribing MHT was acceptable, they prescribed upon approval from the patient’s cancer-treating team.

...if they come in and they’ve got flashes, if it’s a cancer that you can use MHT for, then it’s pretty straightforward, I can give them the MHT… if someone’s got some bladder or vaginal symptoms after an examination, we use that. And then even in breast cancer patients, I would liaise with the surgeon about using Ovestin [vaginal estrogen]. I think that they need to be able to maintain their sex life, their bladder and vaginal function. [Primary Care Physician 1, MSAC specialist]

##### Patient-informed evaluation of treatment success

In the absence of structured assessment tools or systematic follow-up data, almost all participants reported relying on patients’ feedback, return consultations, and clinical judgement to determine whether an intervention was successful.

I don’t know whether they get better because it’s just time, to be honest, I don’t know… [Primary Care Physician 3]

Participants mentioned an overall reduction in patients’ symptom burden, but emphasized that managing patients’ expectations was fundamental, noting that it is highly unlikely for a patient to have complete symptom resolution.

...most of them respond pretty well to the treatment that I have given them. I haven’t had anyone that I’ve needed to refer to a menopause specialist, because we haven’t been able to manage it. [Gynaecological Oncologist 1]

Most participants stated that MHT was most consistently associated with substantial symptom relief, while non-hormonal treatments (gabapentin, oxybutynin, and venlafaxine) and non-pharmacological strategies (CBT, and acupuncture) were beneficial for some patients but ineffective or poorly tolerated for others.

…those on MHT usually get relief, they may not get 100% relief, but it’s around the 90% mark…with some of our non-hormonal treatments, it’s probably closer to about 60% relief, for those women, the symptoms are still there, but they’re not as severe or frequent and they can tolerate them more…I’ve also had a number of patients use that resource [CBT] and had a lot of benefit. [Primary Care Physician 5, MSAC specialist]

#### Care constrained by uncertainty and risk

Participants reflected on the constraints that limit HCPs’ ability to provide comprehensive care for women experiencing menopause after cancer. These limits were not structural or logistical; they were also shaped by the expertise and the perceived risks associated with specific interventions.

##### Expertise and capacity constraints

Participants described variable confidence and competence across HCP groups as shaping care delivery. Some participants perceived that primary care physicians, in particular, sometimes have limited knowledge in managing menopause after cancer.

…a lot of GPs, a lot of oncologists find this area very difficult…I get calls from GPs about patients who have been discharged…I do feel that GPs do struggle in the community with patients... [Medical oncology Nurse Practitioner 1]

Some participants reflected on their limits, including gaps in training, time constraints, and scope of practice constraints, which affected the care they could provide.

…It’s a more comprehensive management of the patient than I have the time or expertise to do…. [Breast Surgeon 1]

Limitations also extended to non-pharmacological interventions such as CBT or clinical hypnosis. Even where evidence supported the efficacy, the absence of trained providers constrained care.

…there is evidence of CBT… the problem then is trying to find someone who can actually deliver that [Primary Care Physician 6]

##### Negotiating risk—“hesitation in prescribing MHT” 

Of the nine participants authorised to prescribe MHT, five, mostly primary care physicians, were hesitant to prescribe it, even when not contraindicated. Participants described their hesitancy as stemming either from ethical concerns (e.g. weighing of potential harm against symptom relief) or from limited confidence and experience in prescribing MHT.

I wouldn’t put anyone on any [MHT], even vaginally…I don’t want them to look back on getting cancer recurrence, and thinking to themselves, if I shouldn’t have taken the X, Y or Z tablet... [Primary Care Physician 4]

## Discussion

To the best of our knowledge, this study is among the first qualitative reports to describe multidisciplinary HCPs’ perspectives on managing menopause after cancer. Key findings include contested views of treatment-induced menopause, how they navigate uncertainty and risk—particularly around MHT—and how service configuration, including limited access to MSAC clinics, shapes care.

For this engaged group, accounts revealed competing stances on whether or not treatment-induced menopause should be understood as inherently severe or comparable to natural menopause. Several studies support the view that treatment-induced menopause is more severe [1, 17, 40–42]. By contrast, a single prospective study of pre-menopausal women undergoing risk-reducing salpingo-oophorectomy (RRSO) found 86% of the women reported mild vasomotor symptoms and 14% moderate or severe symptoms [43]. When compared with population-based data from women with natural menopause [44, 45], the authors concluded that severe vasomotor symptoms could occur after RRSO, but may not be worse than natural menopause [43]. This finding may explain why some participants challenged the notion that treatment-induced menopause is inevitably more severe. This lack of a shared conceptual framework for symptom severity, in our opinion, could impede consistent and proactive care, as evidenced by our HCP-dependent variations in symptom assessment and validation, management strategies, and referral practices.

Most participants, regardless of their position, appeared to provide patient-centred care—a gold-standard approach known to enhance patients’ satisfaction [46, 47]. Our participants were an engaged group; hence, this finding may not reflect wider practices. Recent studies suggest that many breast cancer survivors perceive some HCPs to dismiss their symptoms and report feeling insufficiently involved in treatment decisions [11, 48, 49], alongside inadequate information provision [12, 48], insufficient treatments [7, 14], and limited support [7, 50], and felt not involved in treatment decisions [11]. While these experiences may not reflect all care settings or providers, they highlight ongoing gaps in menopause care experienced by at least some breast cancer survivors. However, these evidence should be interpreted in light of methodological limitations common to survey and qualitative research in this area, including self-selected samples and the potential over-representation of women experiencing higher symptom burden or dissatisfaction with care. Consistent with survivor accounts, some of our participants, and those in previous studies [25–27], highlighted that limited knowledge—their own and that of other HCPs—constrained the delivery of “best” care.

While training gaps may apply to particular HCP groups whose menopausal management is not a core role, we believe HCPs should possess a baseline level of expertise to address common symptoms. Our data show what is possible when HCPs are engaged and resourced. We propose education initiatives (e.g. menopause societies or specialized-led workshops and webinars) to support non-specialized HCPs in improving their knowledge and promoting more consistent care. We also suggest integrating menopause education into medical school curricula to help establish this baseline competency among future providers [51].

Multidisciplinary collaboration and use of referral pathways were essential—seen as good practice and a strategy in areas of perceived clinical and medicolegal risk. These practices observed in our sample align with the National Institute for Health and Care Excellence (NICE) UK (2024 update) guidelines, which recommend referral to HCPs with expertise in menopause [52]. Participants particularly referred to MSAC clinics, reported to benefit 83% of cancer survivors, with improvements in hot flushes, sexual interest, and sleep [14, 53]. However, access remains limited due to scarcity—there are only two MSAC clinics in Australia [3, 54]. In a community-based study among breast cancer survivors, only 3.8% attended an MSAC clinic for symptom management [7]. We suggest that policymakers and health services consider establishing and/or funding additional MSAC clinics (or similar specialist services) globally, to strengthen rehabilitation and supportive care after cancer.

Our participants predominantly used non-medical and non-hormonal therapies as first-line management, consistent with the NICE UK guidelines (2024 update) [52] and reserved MHT as a second-line option, in line with a recent study [1]. Also consistent with the NICE guideline [52], and a previous study [1], is the collaborative decision regarding MHT, in which the patient’s oncology team, relevant specialists, and the woman were consulted. Nevertheless, some of our participants expressed reluctance to prescribe MHT, and gaps in HCPs’ knowledge and confidence to prescribe it, a finding echoed in prior studies [19, 27]. Another study found that deficient knowledge of the true evidence base, together with limited training on the efficacy, safety, and benefit/risk ratio of personalized MHT, was a barrier to prescribing MHT [55]. To address these barriers, we recommend HCPs with an interest in managing menopause in cancer survivors consult existing guidelines, for instance, the NICE guidelines [52], those from the Royal Australian and New Zealand College of Obstetricians and Gynaecologists (RANZCOG) [56], and the International Menopause Society (IMS) (2025) [57], and a recent *Lancet* review [1], which offers recommendations and frameworks to support HCPs in making informed decisions around MHT.

Beyond HCP-level factors, misleading media information, women’s misconceptions about MHT [55, 58], perceived or overestimated risk of cancer recurrence [59, 60], and medicolegal concerns linked to the Women’s Health Initiative (WHI) findings [61] may reinforce hesitancy and reduce the likelihood that HCPs discuss MHT as a treatment option for eligible cancer survivors. For instance, up to one-half of breast cancer survivors with debilitating symptoms may choose to accept MHT, if the benefits of improved QoL and reduced risk of osteoporosis outweigh a small increased risk of cancer recurrence [11, 62–64]. This evidence is particularly relevant given that MHT is generally avoided in breast cancers due to risk of recurrence, especially in oestrogen-receptor-positive; exceptions may be considered when a patient’s quality of life is substantially affected and the risk of cancer recurrence is assessed to be low following thorough discussions and shared decision-making [1].

A further challenge identified was the infrequent use of structured assessment tools and systematic follow-up to evaluate treatment outcomes. Our findings suggest that integrating validated patient-reported outcome measures (PROMs)—such as the Hot Flush Related Daily Interference Scale (HFRDIS), Day-to-Day Impact of Vaginal Aging (DIVA), and Patient-Reported Outcomes Measurement Information System (PROMIS Scale v2.0) [65, 66]—into routine practice could enable more tailored interventions, improve reliability of outcome evaluation, and generate robust data to inform future research. Prior studies have shown an association between PROM use and improvements in symptom management and quality of life in post-survivorship care [67].

### Study strengths and limitations

Strengths of our study include purposive sampling of HCPs across diverse clinical disciplines and Australian states to ensure regional representation. As a qualitative study in Australia, findings are not generalizable, but core themes identified may nonetheless resonate with HCPs in other high-income settings, although contextual differences in healthcare systems and resource availability may shape how their perspectives are expressed. The sample comprised an engaged group interested in menopause after cancer, including HCPs working in or closely with specialist services; it therefore may not represent the wider workforce. Finally, interviews combined prescribers (e.g. primary care physicians and gynaecologists) and non-prescribers (e.g. breast care nurses and pharmacists), which should be considered when interpreting the results.

### Future research

Future research should prioritize four interlinked areas: broaden engagement to include less-engaged HCPs and directly compare clinician accounts with women’s experiences; test targeted education and decision-support interventions for safe MHT prescribing and selection of non-hormonal options in cancer survivorship; conduct health services research to evaluate scalable models to expand specialist input (e.g. stepped-care model), assessing feasibility, clinical impact, and cost-effectiveness; and also examine PROMs within MSAC management to generate real-world outcome data to inform service design.

## Conclusion

Our findings show that our highly engaged HCPs recognize the burden of menopausal symptoms after cancer. They reported adopting a multidisciplinary approach, with shared decision-making and provided patient-centred care. Participants used varied treatment strategies but prioritized low-risk treatments. However, some had inadequate knowledge and variable confidence in prescribing MHT, and limited capacity, and time constraints were barriers to providing optimal care. Targeted education and training, decision-support resources, and expanded access to the specialist MSAC clinic (including telehealth) could improve equitable care.

## Supplementary Information

Below is the link to the electronic supplementary material.

Supplementary file1 (DOCX 38 kb)

## Data Availability

The datasets utilized and/or analyzed as part of this study can be obtained from the corresponding author upon reasonable request.
