# Supplementary material for: Managing menopause after cancer: a qualitative analysis of healthcare professionals’ perspectives
Source: J Cancer Surviv. 2026 May 18;20(Suppl 2):2064–77. doi: 10.1007/s11764-026-02042-0 (PMC13744453; doi:10.1007/s11764-026-02042-0)
Supplement: Supplementary file 1 [file 11764_2026_2042_MOESM1_ESM.docx]

## Semi-structured Qualitative Interview Questions

1. Please can you describe what your role has been in the management of cancer survivors’ menopausal symptoms?

2. In the survey, you stated that you [do, do not, seem to be the same *or* unsure– *refer to question 21*] tend to notice that cancer survivors experience more severe menopausal symptom bother compared to other women with naturally induced menopause. Can you explain this in more detail please?

(**Prompt:** What symptoms do you see more often? How does it impact these women’s lives more compared to women who haven’t undergone menopause?)

3. What common management options do you offer these patients? Of course, the answer to this question depends on the symptoms. But, if possible, what generally do you offer/see get offered to patients?

**(Prompt:** Perhaps it would help to talk through your thought process when you have a patient sitting in front of you saying that they experience hot flushes. What would you do next? How would you determine how to manage her symptoms?)

4. Have you found your patients are experiencing relief from these treatments?
5. Referral to specialists:

**If GP or oncologist or gynaecologist or gynaecological oncologist or surgeon or pharmacist:** how often do you refer these patients to a specialist, and which specialists? Do you have a threshold of symptom-degree before you refer?

**If practice or oncology nurse:** how often do you see these patients get referred to a specialist?

**If menopausal specialist:** as you have patients referred to you, do you think they are over or under referred to your clinic?

**Closing up:**

6. Is there anything else you would like to tell me or ask me?

Thank you for your participation in this study. We will soon send you your remuneration via email.
